# Effect of different forms of tobacco on the oral microbiome in healthy adults: a systematic review

**DOI:** 10.3389/froh.2024.1310334

**Published:** 2024-02-20

**Authors:** Nikitha Lalindri Mareena Senaratne, Cheng Yung on, Naresh Yedthare Shetty, Divya Gopinath

**Affiliations:** ^1^ School of Medicine, International Medical University, Kuala Lumpur, Malaysia; ^2^ Faculty of Medicine and Health, UNSW, Sydney, NSW, Australia; ^3^ Sungai Rengit Dental Clinic, Johor Health Department, Ministry of Health Malaysia, Kota Tinggi, Malaysia; ^4^ Clinical Sciences Department, Ajman University, Ajman, United Arab Emirates; ^5^ Centre of Medical and Bio-Allied Health Sciences Research, Ajman University, Ajman, United Arab Emirates; ^6^ Basic Medical and Dental Sciences Department, Ajman University, Ajman, United Arab Emirates

**Keywords:** microbiome, oral, tobacco, smokers, microbiota, chewers

## Abstract

**Objective:**

The study aimed to evaluate the impact of tobacco use on the composition and functions of the oral microbiome in healthy adult humans.

**Methods:**

We conducted a systematic search on PubMed, Web of Science, and Cinhal databases for literature published until 15 December 2023, to identify studies that have evaluated the oral microbiome with culture-independent next-generation techniques comparing the oral microbiome of tobacco users and non-users. The search followed the PECO format. The outcomes included changes in microbial diversity and abundance of microbial taxa. The quality assessment was performed using the Newcastle–Ottawa Scale (NOS) (PROSPERO ID CRD42022340151).

**Results:**

Out of 2,435 articles screened, 36 articles satisfied the eligibility criteria and were selected for full-text review. Despite differences in design, quality, and population characteristics, most studies reported an increase in bacterial diversity and richness in tobacco users. The most notable bacterial taxa enriched in users were *Fusobacteria* and *Actinobacteria* at the phylum level and *Streptococcus*, *Prevotella*, and *Veillonella* at the genus level. At the functional level, more similarities could be noted; *amino acid metabolism* and *xenobiotic biodegradation pathways* were increased in tobacco users compared to non-users. Most of the studies were of good quality on the NOS scale.

**Conclusion:**

Tobacco smoking influences oral microbial community harmony, and it shows a definitive shift towards a proinflammatory milieu. Heterogeneities were detected due to sampling and other methodological differences, emphasizing the need for greater quality research using standardized methods and reporting.

**Systematic Review Registration:**

CRD42022340151.

## 1 Introduction

The human oral cavity harbors a diverse microbial community comprising over 700 species of bacteria or phylotypes that play a commensal role in protecting oral and systemic health (1). These diverse species have been identified by cultivation or the advancing culture in-dependent molecular approaches (1). These species attach and form biofilms on the mouth's soft and hard tissue surfaces in a structurally organized matrix, inducing a dynamic equilibrium with the immune-inflammatory response of the host (2). The human oral cavity serves as one of the major gateways to the respiratory tract, thus giving microorganisms the substantial prospect of invading these sites (3). Despite the similarities between the core microbial composition within the oral cavities, the type of species may vary depending on diet and nutrition, genetic susceptibility, antibiotic usage, hormonal factors, tobacco and alcohol exposure, and recurrent pathogenic infections of the host (4). This disturbance to the equilibrium results in oral dysbiosis altering oral and systemic health through several pathophysiological processes linked to disease (5). Dysbiosis has reportedly been involved in oral diseases such as periodontitis, gingivitis, and oral cancer (6–8).

The emergence of new genomic technology including next-generation sequencing, has led to the identification of resident bacterial populations in almost all organs and systems of the body, and has sparked an increased interest in the microbiota among researchers. These next generation sequencing helped to reveal the complex nature of the oral microbiome community, which could not be revealed by culture methods and traditional Sanger sequencing methods as less abundant and non-cultivable microbes of the population are often overlooked, which jeopardizes the accuracy of the detailed account of the microbial community (9).

Recent studies show that despite a global decline in tobacco consumption, tobacco use is exponentially rising in parts of the world, leading to a consequential public health concern (10). Tobacco smoke comprises numerous toxicants that come into direct contact with the bacteria in the oral cavity, disrupting the microbial ecology of the mouth. These toxic compounds cause cellular injury and cell death, including N-nitrosamines and polycyclic aromatic hydrocarbons blocking DNA repair and initiating tumorigenesis (11). Smoking has been shown to cause the loss of beneficial oral species, leading to pathogenic alterations by interacting with various host cells and extracellular matrix components, ultimately leading to the risk of disease development (12). This alteration increases the local density of the bacterial pathogens or decreases the prevalence of other bacteria (13, 14). Emerging evidence on the effects of smokeless tobacco on the composition of the oral microbiota in humans suggests it leads to a pro-inflammatory milieu in the oral microenvironment, further leading to diseases (15). To date, the literature on the effects of tobacco use on the oral microbiome in humans has not been systematically evaluated. Therefore, we carried out a systematic review as a first attempt to characterize the impact of tobacco use on the oral microbiome profile in healthy adults and to compare the differences in the oral microbiome profile of tobacco users with non-users. It also aims to highlight the potential effects of smoking on the host's health by analyzing the available data regarding the relationship between the human oral microbiome and tobacco use.

## 2 Material and methods

### 2.1 Search strategy

A systematic review was conducted to answer the question: “Is the oral microbiome profile of tobacco users different from non-users?” The present systematic review was registered in the International Prospective Register of Systematic Reviews (PROSPERO) under CRD42022340151) The systematic literature search was performed to identify published studies until Dec 2023 examining the oral microbial community in tobacco users in comparison to controls using broad MeSH terms and other related keywords. The search was performed independently by two investigators (NS and CY). The electronic databases used are PubMed, Web of science and CINHAL. The search was carried out using the specific key keywords with the use of Boolean operators “OR” and “AND.” The search strategy and output for each database is provided as Supplementary Tables S1–S3. Following the elimination of duplicates, the titles and abstracts were evaluated in accordance with the preset eligibility criteria as provided below to determine whether or not they should be included for additional full-text reading. Two independent investigators (NS and CY) scanned the titles and corresponding abstracts. If the abstract clearly indicated what was included or excluded, the record was read in its entirety. In the event that the findings of the two investigators disagreed, DG, the third investigator, was consulted. We manually examined the reference lists of the included publications to find any potentially relevant articles that could be included. The systematic review follows in accordance with the PRISMA (Preferred Reporting Items for Systematic Reviews and Meta-Analyses) guidelines (16).

### 2.2 Eligibility criteria

Inclusion criteria

Cross-sectional or prospective observational studies that compared the oral microbiome analyzed with culture-independent next-generation techniques from tobacco users, including cigarettes, water pipes, smokeless, and other forms of tobacco in comparison to healthy controls were included. The detailed PECO (Population, Exposure, Control, and Outcome) scheme followed is below:

Population: Human adults using tobacco

Exposure: Use of any form of tobacco

Control: Non-users

Outcome: Changes in microbial diversity and abundance of various microbial taxa

Type of studies: Cross-sectional or prospective observational studies that utilized culture-independent next-generation techniques without date limitation.

Exclusion criteria

The studies which did not fit into the inclusion criteria were excluded.

Studies utilizing culture techniques, studies on diseased populations like periodontitis or caries, which can have an impact on the oral microbiome, animal studies, and studies on e-cigarettes were excluded. Further narrative reviews, systematic reviews, conference reports, and letters to the editor were excluded. The literature search was limited to the English Language.

### 2.3 Data Extraction

Data was extracted from the selected articles through a separate full-text review by two reviewers. The following study characteristics were extracted from each article: author name, year of publication, study design, sample size, age and gender distribution, type of tobacco, exposure assessment, and significant changes in oral microbial diversity and abundances of taxa.

### 2.4 Quality assessment

The quality assessment for the included studies was performed independently by two reviewers (NS and CY) using the Newcastle–Ottawa Quality Assessment Scale (17). If there is any discrepancy, then the third author was consulted (DG) and the discrepancy was resolved. This instrument incorporates three separate domains: selection, comparability, and outcomes. The selection domain involves the assessment of four items; comparability has one item, and outcomes include three items. The selected article will receive one star in each item if acceptable, thus obtaining a maximum of four in the selection domain, one in the comparability domain, and three in the outcome domain.

## 3 Results

### 3.1 General study characteristics

**Figure 1:**
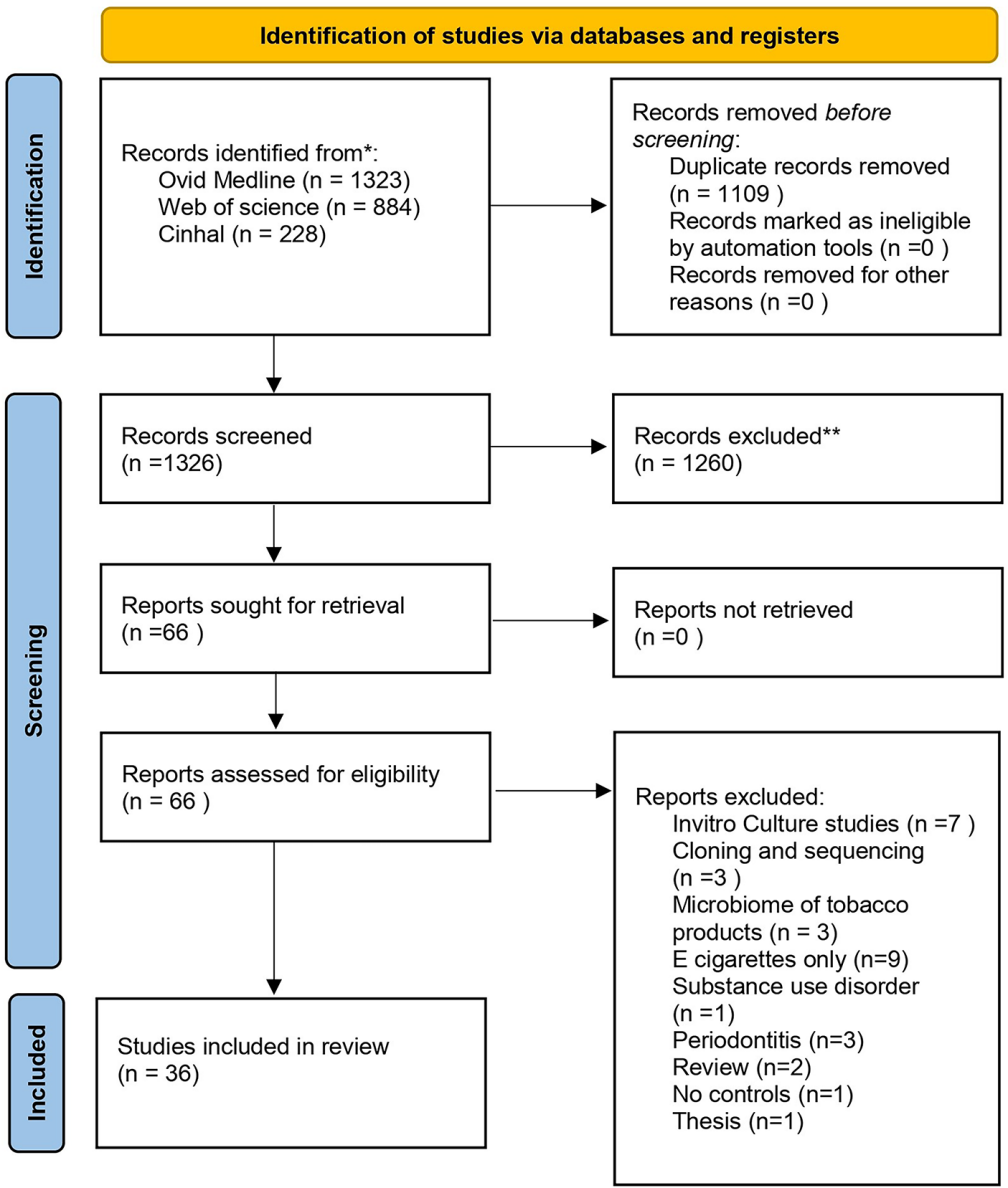
The prisma flow chart.

The search yielded 2,435 records from the three databases, of which 1,109 were excluded altogether due to duplicates. Screening of articles by title and abstract and reviewing of full text resulted in 36 eligible articles for full text (Figure 1). Of the 36 articles, nine were from the United States of America (18–26), six were from India (15, 27–31), five were from the United Arab Emirates (32–36),three were from China (37–39), two from Japan (40, 41) and others including Brazil (42), Jordan (43), Hungary (44), Croatia (45), Iran (46), Germany (47), Denmark (48), Italy (49), Sudan (50), Ireland (51) and Korea (52).

The study design followed cross-sectional studies with a sample size ranging from 22 to 1,616. Most studies were conducted on cigarette users, except seven studies that focused on smokeless tobacco products including chewing tobacco (20, 27–30, 34, 50). The 16s rRNA gene sequencing was the most commonly used methodology except for three studies that used shotgun metagenomic gene techniques (28, 33, 44). A common trait seen in most studies was screening for antibiotic usage before sampling and for the presence of chronic or oral illnesses. Further, some studies also included decisive factors that can influence the microbiome, including alcohol consumption, BMI, and diet, into consideration for profiling of the subjects (25, 27, 31, 38). Sample collection types include saliva, oral and buccal swabs, oral rinses, supragingival, subgingival and tongue scrapes, and mouthwashes. The detailed characteristics are provided in Table 1 (42–52). All controls were deemed healthy except for one study that acquired control subjects from cancer cohorts (19).

**Table 1:** Characteristics of the selected studies.

No	Author, Country, Year	Study Design	Sample Characteristics	Type of tobacco	Amount of Exposure—Assessment	Methodology	Statistical Adjustments
1	Thomas et al. (42), Brazil, 2014	Cross-sectional	*N* = 22 6 active smokers 7 smokers and drinkers 9 controls	Cigarette	Smokers—20 cigarettes/day for past 10 years	V1 region of 16s rRNA gene sequencing	Subject with cancer, use of antibiotics within last 3 months, comorbidities, presence of oral lesions were excluded
2	Mason et al. (18), USA, 2015	Cross-sectional	*N* = 200 100 current smokers 100 never smokers	Not specified	N/A	16s rRNA sequencing	Diabetes, HIV, pregnancy, immunosuppressants, bisphosphonates, steroids, antibiotics, current orthodontic therapy, or professional dental cleaning within 3 months and pre-treatment using antibiotic were excluded
3	Wu et al. (19) USA, 2016	Cross-sectional	*N* = 1,204 112 current smokers 471 former smokers 521 never Smokers	Cigarette	Assessed but not specified	V3 to V4 regions of 16s rRNA gene sequencing	No cancer prior to sampling Age and sex was adjusted
4	Hernandez et al. (20) USA, 2017	Cross-sectional	*N* = 122 64 current chewers 37 former chewers 21 non chewers	Chewing tobacco	Long term chewers: >10 years	V3 to V5 region of 16s rRNA gene sequencing	No history of oral cancer
5	Yu et al. (21) USA, 2017	Cross-sectional	*N* = 43 23 current smokers 20 never smokers	Cigarette	Smokers: >100 cigarettes in a life time	V3 to V4 regions of 16s rRNA gene sequencing	Age, gender, race, antibiotic usage or professional dental cleaning within the last 3 months or diagnosed with periodontal disease or cancer or losing >1 tooth were excluded
6	Rodríguez- Rabassa et al. (22) USA, 2018	Cross-sectional	*N* = 34 15 non-smokers 18 current smokers	Cigarette	Assessed but not specified	V3 to V4 regions of 16s rRNA gene sequencing	Age, sex, race, education level (high school/college) was adjusted
7	Stewart et al. (23) USA, 2018	Cross-sectional	*N* = 30 10 e-cigarette users 10 tobacco smokers 10 controls	E-cigarette Cigarette	E-cigarette—daily use for at least 6 months Tobacco smokers ≥4 and ≥10 cigarettes per day	V4 region of 16s rRNA gene sequencing	Sex, age, diet, height/weight and race adjusted
8	Vallès et al. (32) UAE, 2018	Cross-sectional	*N* = 330 105 smokers 225 non-smokers	Cigarette Dokha Shisha	Self-reported	16s rRNA gene sequencing	Tobacco smoke exposure cut-off concentration of 200 ng/ml
9	Beghini et al. (24) USA, 2019	Cross-sectional	*N* = 297 90 current smokers 45 never smokers 45 former smokers 38 non-smokers with second hand exposure 79 alternative smokers	Cigarette E-cigarette Hookah Cigar Cigarillo	Current smokers: >100 cigarettes. Never smokers: <100 cigarettes, serum cotinine <0.05 ng/ml Former smokers: >100 cigarettes, serum cotinine <0.05 ng/ml Non-smokers: serum cotinine 1–14 ng/ml	V4 region of 16s rRNA gene sequencing	Subjects who smoked in the last 5 days were excluded
10	Lin et al. (26) USA, 2019	Cross-sectional	*N* = 60 30 smokers 30 non-smokers	Cigarette	N/A	16s rNA sequencing	Subjects not treated for nicotine use, serious medical or psychiatric conditions, use of illicit drugs or on insulin or oral hypoglycaemic medications were excluded. Age and gender adjusted
11	Yang et al. (25) USA, 2019	Cross-sectional	*N* = 1,616 592 current smokers 477 former smokers 547 never smokers	Cigarette	N/A	V4 region of 16s rRNA gene sequencing	Age, sex, race, body mass index, alcohol consumption, total energy intake, oral and disease status adjusted.
12	Al Bataineh et al. (33) UAE, 2020	Cross sectional	*N* = 105 55 smokers 50 non-smokers	Cigarette	Cigarette smokers: ≥5 years	Shotgun metagenomic sequencing	Antibiotic or prescribed probiotic use in the past three months, and those with pre-existing respiratory illness such as asthma and chronic obstructive pulmonary disease excluded
13	Al-Zyoud et al. (43) Jordan, 2020	Cross-sectional	*N* = 100 49 smokers 51 non-smokers	Cigarette	Smokes at least 1 cigarette per day	V3 to V4 regions of 16s rRNA gene sequencing	Antibiotic free for the last three months No chronic oral diseases
14	Halboub et al. (34) UAE, 2020	Cross-sectional	*N* = 52 29 smokers 23 non-smokers	Smokeless tobacco (Shammah)	Daily for at least 1 year without cessation	V1 to V3 regions of 16s rRNA gene sequencing	Subjects with moderate to severe gingivitis or periodontitis, history of antibiotic, antifungal or steroids use and periodontal treatment, including prophylaxis in the last 3 months were excluded
15	Sato et al. (40) Japan, 2020	Cross-sectional	*N* = 657 364 never smokers 129 former smokers 144 current smokers	Cigarette	N//A	V3 to V4 regions of 16s rRNA gene sequencing	Subjects on oral antimicrobials or steroids, low GFR rate, on anti-hypertensive drugs, hypoglycaemic agents or probiotics were excluded
16	Wirth et al. (44) Hungary, 2020	Cross-sectional	*N* = 22 11 smokers 11 non-smokers	Cigarette	Cigarette smokers: ≥20 cigarettes/pack year	Shotgun metagenomic sequencing—real time PCR	Chronic illnesses and treatment with antibiotics for at least 6 months prior to sampling were excluded
17	Bašić et al. (45) Croatia, 2021	Cross-sectional	*N* = 64 32 smokers 32 non-smokers	Cigarette	Smokers—1 pack/day	MALDI-TOF mass spectrometry	Presence of periodontitis, systemic diseases, mediation, pregnancy, less than 20 teeth, use of antibiotics six months prior and periodontal or orthodontic therapy use was excluded.
18	Al Kawas et al. (35) UAE, 2021	Cross-sectional	*N* = 40 10 controls 10 cigarettes smokers 10 shisha smokers 10 medwakh	Cigarette Shisha Medwakh	N/A	16s rRNA gene sequencing	Patients who were currently receiving orthodontic treatment and those who had any periodontal treatment, antibiotics, or steroid therapy in the last 3 months were excluded
19	Jia et al. (37) China, 2021	Cross-sectional	*N* = 316	Cigarette	Current Smokers: one cigarette every 1–3 days for 1 year Former Smokers: no smoking for a year	16s rRNA gene sequencing	Amplicon sequence variants in fewer than three samples and with abundances less than five were excluded
20	Li et al. (38) China, 2021	Cross-sectional	*N* = 76 16 smokers 60 non-smokers	Cigarette	Not specified	V4 region of 16s rRNA gene sequencing	No oesophageal cancer, low-grade dysplasia (LGD), high-grade dysplasia (HGD) Age, gender, BMI adjusted
21	Srivastava et al. (27) India, 2021	Cross-sectional	*N* = 40 20 smokers 10 non-smokers	Smokeless tobacco	Smokers—>5 years with 25 g of SLT product intake a week	V3 region of 16s rRNA gene sequencing	Subjects who were alcoholic and on any medications or antibiotics were excluded
22	Wu et al. (46) Iran, 2021	Cross-sectional	*N* = 558 120 cigarette only users 120 never users 49 opium only users	Cigarette	N/A	16s rRNA gene sequencing	Subjects who had a normal pancreas at the endoscopic ultrasonography exam, aged 40 years or older, no history of liver or renal failure or cancer, no consumption of a special diet, and did not develop pancreatic disease or any cancer within one year of the initial visit
23	Al-Marzooq et al. (36) UAE, 2022	Cross-sectional	*N* = 40 10 control 10 cigarette smokers 10 shisha smokers 10 medwakh smokers	Cigarette Shisha Medwakh	N/A	16s rRNA gene sequencing	Subjects who smoked more than one type of tobacco and had less than 10 teeth were excluded
24	Gopinath et al. (15) India, 2022	Cross-sectional	*N* = 44 17 smokers 14 smokeless tobacco users 14 non-smokers	Cigarettes/Bidis Smokeless tobacco	Tobacco use—1–12 years	16s rRNA gene sequencing	Subjects to refrain from smoking, drinking and eating 30 min before sample collection
25	Pfeiffer et al. (47) Germany, 2022	Cross-sectional	*N* = 58 30 smokers 6 ex-smokers 10 never-smokers	Cigarette	Long term Smokers: ≥10 daily cigarettes & ≥10 pack years Short term smokers: ≥10 daily cigarettes & <10 pack years Mild smokers: <10 daily cigarettes & <5 pack years	16s rRNA gene sequencing	N/A
26	Poulsen et al. (48) 2022, Denmark	Cross-sectional	*N* = 746 350 ex-smokers	N/A	N/A	16s rRNA gene sequencing	N/A
27	Sharma. (28) 2022, India	Cross-sectional	–	Chewing tobacco	N/A	Metagenomic sequencing	N/A
28	Suzuki et al. (41) Japan, 2022	Cross-sectional	*N* = 50 (39M, 11F) 18 smokers 32 non-smokers	Cigarette	Smokers: ≥100 cigarettes after initiation of smoking	16s rRNA gene sequencing	Subjects who scored more than 0 for bleeding on probing and probing pocket depth were excluded
29	Antonello et al. (49) Italy, 2023	Cross-sectional	*N* = 1601 720 current/former smokers 881 non-smokers	Cigarette	Current smokers—reduced daily smoking intensity one month prior	V4 region of 16s rRNA sequencing	Sex, age and number of teeth were adjusted Use of antibiotics for last 3 months and missing date on number of teeth were excluded
30	Bahuguna et al. (29) India, 2023	Cross-sectional	*N* = 22 9 chewers 9 non-chewers 4 occasional/previous chewers	Chewing tobacco	Chewers—habitual individuals Occasional/previous chewers—once in a couple of months/previous history of chewing	16s rRNA sequencing	N/A
31	Huang et al. (39) China, 2023	Cross-sectional	*N* = 587 111 smokers 467 non-smokers	Cigarette	Pack years but not specified	16s rRNA gene sequencing	Subject with disease and microbial features of cardio metabolic risk factors were excluded
32	Sami et al. (50) Sudan, 2023	Cross-sectional	*N* = 78 47 smokers 32 non smokers	Smokeless tobacco (toombak)	N/A	16s rRNA sequencing	Absence of periodontal disease and dental infection, controlled caries mouth, use of antibiotics the past 3 months
33	Sawant et al. (30) India, 2023	Cross-sectional	*N* = 120 40 controls 40 long term tobacco chewers 40 oral cancer patients	Chewing tobacco	Chewing tobacco—≥5 years	16s rRNA gene sequencing	Use of antibiotic treatment for one week prior, previous oncotherapy, medically compromised and edentulous subjects were excluded
34	Galvin et al. (51) Ireland, 2023	Cross-sectional	*N* = 322 148 current smokers	Cigarette	N/A	V1toV3 region of 16s rRNA gene	Use of antibiotics or topical steroids intra-orally in the past 3 months, patients with diabetes mellitus, chron's disease, ulcerative colitis, current viral infection and history of gastrointestinal malignancy were excluded
35	Yadav et al. (31) India, 2023	Cross-sectional	*N* = 50	Cigarette	Smokers—past 5 years	V3 to V4 region of 16s rRNA gene sequencing	Ex-smokers and subjects who both smoked and consumed alcohol were excluded
36	Yu et al. (52) Korea, 2024	Cross-sectional	*N* = 43	Not specified	N/A	16s rRNA gene sequencing	Use of antibiotics for one month and food or water intake two hours prior sample collection was restricted.

### 3.2 Diversity and richness analysis

As displayed in Table 2 (42–52), all included studies except five assessed microbial diversity and richness (23, 28, 31, 38, 45). Five studies reported no difference in diversity difference between the smokers and control groups (34, 36, 41, 49, 52). Four studies (21, 26, 40, 42) reported lower diversity and richness in smokers. The rest of the studies concluded that the richness and phylogenetic biodiversity of smokers or tobacco users were significantly different or higher than non-users or former users.

**Table 2:** Characteristics of oral microbiome from the selected studies.

No	Author, Country, Year	Sample Type	Age (Range/Mean/Median)	Other clinical features studied	Results: Diversity and Richness	Bacterial taxa associated with
1	Thomas et al. (42) Brazil, 2014	Oral swab	Overall—>40 years Smokers—56.67 ± 2.49 Smokers/drinkers—59.86 ± 3.39 Control—58.11 ± 8.28	Effects of chronic alcohol use on the oral micro biome	Decrease in species richness in smokers	Smokers had significant increases in *Prevotella* and *Capnocytophaga* and reductions in *Granulicatella*, *Staphylococcus*, *Peptostreptococcus* and *Gemella.* Smokers/drinkers had lower abundances of *Fusobacteria*
2	Mason et al. (18) USA, 2015	Subgingival plaques	Overall—21–40 years Never smokers—27.0 ± 5.3Current smokers—28.25 ± 3.5	Not assessed	Higher diversity in smokers	The subgingival microbiome of smokers was enriched with *Fusobacterium nucleatum*, *S.mutans* and *Lactobacillus salivarius* and lower levels of *Streptococcus sanguinis*, *S.oralis* and *Hemophilus parainfluenzae*
3	Wu et al. (19) USA, 2016	Oral rinse	Current Smokers—68.82 Former Smokers—70.71 Never smokers—70.53	Prospective development of head and neck cancer and pancreatic cancer	Current smokers had an increased diversity	Current smokers had decreased abundance of phylum *Proteobacteria* Genera *Peptostreptococcus*, *Capnocytophaga*, *and Leptotrichia* were depleted. In contrast, *Atopobium* and *Streptococcus* were enriched in current smokers compared with never smokers
4	Hernandez et al. (20) USA, 2017	Oral swab Saliva	Overall—18–60 + years	Body mass index	Alpha diversity lower in current chewers	Current chewers had elevated levels of *Streptococcus infantis* and lower levels *Actinomyces* and *Streptococcus genera*. Long-term chewers had reduced levels of *Parascardovia* and Streptococcus. Chewers with oral lesions had elevated levels of *Oribacterium*, *Actinomyces*, and *Streptococcus*
5	Yu et al. (21) USA, 2017	Subgingival plaque scrapes, saliva, oral swab	Assessed but not specified	N/A	Alpha diversity was lower in smokers than in non-smokers in the buccal mucosa	*Streptococcus* was the most abundant across all types of oral samples followed by *Veillonella*
6	Rodríguez- Rabassa et al. (22) USA, 2018	Saliva	Smokers—54 Non-smokers—34	Cytokine levels and symptoms of depression	Beta diversity between smokers and non-smokers were *p* < 0.05	*Proteobacteria*, *Firmicutes*, *Bacteroidetes*, *Fusobacteria and Actinobacteria* dominated in smoker samples
7	Stewart et al. (23) USA, 2018	Saliva Buccal swab	Control—31 (28–36) E-cigarette—29 (24–37) Tobacco smoker—35 (30–45)	N/A	N/A	Cigarette users were associated with significantly lower abundance of *Bacteroides and Prevotella* compared to EC users and non-smokers
8	Vallès et al. (32) UAE, 2018	Mouthwash	Smokers—32.4 Non-smokers—33.1 Cigarette—36.4 Dokha—30.8 Shisha—35.7	N/A	Tobacco users had higher diversity	*Cyanobacteria*, *SR1*, *Cyanobacteria) and BD1*–*5 (GN02)* were all depleted in smokers *Actinobacillus* depletion was consistently observed across all four types of tobacco
9	Beghini et al. (24) USA, 2019	Oral rinse	Overall—>18 years	N/A	Difference in beta diversity between current smokers and never smokers. No alpha diversity difference	*Streptococcus* and P*revotella* was the predominant genera, while *proteobacteria* was less abundant in smokers Phyla *Actinobacteria*, *Firmicutes* and *Proteobacteria* were more abundant in alternative smokers. In hookah users, *Porphyromonas*, *Leptotrichia*, *Streptobacillus* and *Fusobacterium* were depleted
10	Lin et al. (26) USA, 2019	Saliva	Overall—37.2 ± 10.65 (21–56 years)	Brain functional connectivity and neurological signalling in smokers, alcohol use identification and marijuana smoking	Decrease of beta diversity in smokers	*Bacteroides*, *Treponema*, *Mycoplasma*, *TG5*, *Actinomyces* spp was abundant in smokers. Depletion of *Lautropia* and *Neisseria* were also seen in smokers
11	Yang et al. (25) USA, 2019	Oral rinse	Current Smokers—53.18 ± 7.90 Former Smokers—59.18 ± 8.49 Never Smokers—55.78 ± 8.88	Body Mass Index	Current smokers had increased diversity	Phylum *Actinobacteria*, *Bifidobacterium* and *Lactobacillus*, were enriched among current-smokers Phylum *Proteobacteria* was depleted in current smokers
12	Al Bataineh et al. (33) UAE, 2020	Buccal swab	Smokers—30.40 Non-smokers—30.30	Nicotine dependence	Heavy smokers had an increase in diversity	Smokers had significant abundance of *Veillonella dispar*, *Prevotella pleuritidis* and *Leptotrichia spp* when compared to non-smokers
13	Al-Zyoud et al. (43) Jordan, 2020	Saliva	23.9 ± 6.20 27.1 ± 7.57	N/A	Higher richness in smokers vs. non-smokers.	*Streptococcus*, *Prevotella*, *and Veillonella* showed significantly elevated levels among smokers and *Neisseria* in non-smokers
14	Halboub et al. (34) UAE, 2020	Tongue scrapes	Overall—20–40 years Smokers—27.34 ± 6.9 years Non-smokes—27.7 ± 7.19 years	N/A	No significant difference in richness or alpha diversity between study groups.	*Firmicutes*, *Actinobacteria*, *Proteobacteria*, *Fusobacteria*, and *Bacteroidetes* were abundant in all samples *Rothia mucilaginosa*, *Streptococcus* sp. *oral taxon 66*, *Actinomyces meyeri*, *Streptococcus vestibularis*, *Streptococcus sanguinis* and *Veilonella* was abundant in smokers
15	Sato et al. (40) Japan, 2020	Tongue coating	Never smokers—49.78 Former smokers—48.03 Current smokers—43.99	N/A	The alpha diversity was lower in current smokers than in never smokers	*Neisseria and Capnocytophaga* were less abundant and *Streptococcus and Megasphaera* were more abundant in current smokers
16	Wirth et al. (44) Hungary, 2020	Saliva	Non-smokers—40 Smokers—41.5	Level of exhaled carbon monoxide and periodontal status	Increase in diversity in the smokers group	*Streptococcus* along with *Prevotella* and *Veillonella* were abundant in both groups *Prevotella* and *Megasphaera* was higher in saliva of current smokers whereas *Neisseria*, *Oribacterium*, *Capnocytophaga* and *Porphyromonas* were reduced
17	Bašić et al. (45) Croatia, 2021	Subgingival plaques	Overall—25–35 years old	N/A	N/A	Prevalence of *Actinomyces odontolyticus* was higher in smokers, while *Streptococcus sanguinis* was lower compared to non-smokers
18	Al Kawas et al. (35) UAE, 2021	Subgingival plaques	Cigarettes—31.9 ± 10.43 Shisha—29.1 ± 12.05 Medwakh—24.1 ± 4.33 Non-smokers—38.5 ± 13.6	Periodontitis	Diversity was equal in all four groups	*Prevotella denticola* and *Treponema sp. OMZ 838* increased abundance in medwakh smokers *Streptococcus sanguinis and Tannerella forsythia* in shisha smokers *Streptococcus mutans* and *Veillonella* in cigarette smokers *Firmicutes* was the most abundant phylum across all groups
19	Jia et al. (37) China, 2021	Saliva	46.98 ± 11.47 46.74 ± 11.16 46.17 ± 11.48	N/A	Difference in alpha diversity between smokers and never smokers	At the genus level, *Actinomyces*, *Oribacterium*, *Atopobium*, *Prevotella*, *Veillonella* and *Campylobacter* were increased in smokers. *Haemophilus*, *Kingella*, *Neisseria*, *Cardiobacterium*, *Aggregatibacter*, *Lautropia*, *Eikenella* and *Moraxella* were significantly depleted in smokers At the species level, *Rothia dentocariosa*, *Prevotella melaninogenica*, *Prevotella pallens*, *Bulleidia moorei* and *Veillonella dispar* were increased in smokers. *Rothia aeria*, *Neisseria oralis*, *Nesseria subfl ava*, *Haemophilus parainfluenzae* and *Actinobacillus parahaemolyticus* were depleted in smokers
20	Li et al. (38) China, 2021	Saliva	Overall—50–70 years	Effect of drinking	N/A for saliva samples	Increase of *Neisseria*, *Prevotella*, *Porphyromonas*, *Fusobacterium*, *and Rothia* and a decrease of *Streptococcus*, *Actinobacillus*, *and Haemophilus* in subjects who smoked
21	Srivastava et al. (27) India, 2021	Oral rinse	Overall—24–58 years	Health Status—diabetic status, systolic BP, BMI	SLT users showed higher richness diversity higher diversity	SLT users had increase abundance of *Fusobacteria*, *Porphyromonas*, *Enterococcus*, *Parvimonas* and *Desulfobulbus*
22	Wu et al. (46) Iran, 2021	Saliva	Cigarette smokers—(82.13 ± 38.55) Cigarette and opium users—(77.80 ± 42.83)Never users—(95.10 ± 44.03).	Use of opium	Lower alpha diversity in cigarette users	*Enterobacteriaceae* was prevalent in cigarette smokers only Abundance of phyla *Actinobacteria*, *Proteobacteria*, *Bacteroidetes*,and *Firmicutes* were noted in smokers and opium users
23	Al-Marzooq et al. (36) UAE, 2022	Supragingival plaque scrapes	18–62 years	Dental carries	No difference	*Firmicutes* was the most abundant phylum in the supragingival plaque samples of all types of tobacco smoking *Proteobacteria* and *Actinobacteria* were significantly abundant in shisha smokers and other types of smokers Overall *Streptococcus* was the most abundant genus
24	Gopinath et al. (15) India, 2022	Buccal swab	Smokers—33.05 Chewers—32.92 Controls—33.69	Levels of carbon monoxide exhaled	Increase in diversity with the use of tobacco	Levels of *Fusobacterium spp*. and *Saccharibacterium spp*. were increased in smokers in comparison to controls. The relative abundance of *Fusobacterium spp.*, *Catonella*, and *Fretibacterium spp*. were significantly higher in smokeless tobacco users
25	Pfeiffer et al. (47) Germany, 2022	Nasal swabs Oropharyngeal swab Bronchoalveolar lavage	N/A	Levels of nicotine and metabolite cotinine	Increase diversity with smoking	*Firmicutes* was relatively higher in abundance in smokers compared to never-smokers *Actinobacteria* was significantly higher in smokers and ex-smokers comparative with never smokers and *Betaproteobacteria* was lower in smokers and ex-smokers in oropharyngeal samples
26	Poulsen et al. (48) 2022, Denmark	Saliva	Overall—68 years	Effect of other lifestyle factors on salivary microbiota	Difference in diversity between smokers and other variables	Genera *Veilonella*, *Streptococcus* and *Rothia* was higher and *Neisseria*, *Haempilus*, *Pophyromonas* and *Actinomyces* in smokers compared to ex-smokers and never smokers
27	Sharma (28) 2022, India	Saliva	N/A	Oral microbiome in oral cancer	N/A	Phylum *Bacteroidetes*, *Firmicutes*, *Proteobacteria*, *Actinobacteria* were abundant in tobacco users
28	Suzuki et al. (41) Japan, 2022	Saliva Tongue samples	Overall—25.6 ± 2.1 (21–31 years) Smokers—26.8 ± 2.4 Non-smokers—25.0 ± 1.6	N/A	No difference	Smoker's saliva was enriched with *Treponema and Selenomonas.* The tongue microbiota from smokers were higher in *Dialister and Atopobium*
29	Antonello et al. (49) Italy, 2023	Saliva	Overall—45 years (18–91 years)	N/A	No changes in alpha diversity	*Firmicutes* were the most abundant, followed by *Bacteroidetes*, *Proteobacteria*, *Actinobacteria* and *Fusobacteria* Increased abundance of *Atopobium*, *Megasphaera*, Fretibacterium, and *Veillonella* when compared to never smokers
30	Bahuguna et al. (29) India, 2023	Oral swab	N/A	N/A	Increased alpha diversity in chewers	*S. pneumoniae*, *S. salivarius*, and *S. Mutans* were increased in oaccasional chewers whereas *Streptococcus* genus was decreased in current chewers. *Prevotella* and *bacteriodes* was increased in chewers
31	Huang et al. (39) China, 2023	Saliva	Smokers—53 years Non-smokers—49 years	Cardiometabolic risk factors	Alpha diversity was higher in smokers	Higher abundance of phyla *Firmicutes* and *Actinobacteriota* *Megasphaera*, *Anaeroglobus*, *Dialister*, Rothia, Atopobium, *Actinomyces*, *Howardella*, and *Romboutsia* and lower relative abundance of the genus *Johnsonella* in smokers was observed
32	Sami (50) Sudan, 2023	Saliva Mucosal and supragingival plaques	Overall—20–70 years	Oral cancer microbiome composition	Alpha diversity was significantly varied between groups	*Staphylococcaceae* and *Corynebacterium_*1 and *Cardiobacterium* was more abundant in smokers *Prevotella*, *Lactobacillus* and *Bifidobacterium* were prominent in non-smokers
33	Sawant et al. (30) India, 2023	Oral rinse	>18 years	N/A	Higher alpha diversity in tobacco chewers and control populations	*Leptotrichia*, *Treponema*, *Lautropia*, *spirochaetes* and *Cardiobacterium* was abundant in tobacco chewers
34	Galvin et al. (51) Ireland, 2023	Oral swab	Overall—≤40 and ≥60 years	Effect of tooth loss, plaque levels and oral hygiene on oral mucosal colonization	No significant changes in alpha diversity	Reduced abundance of *Neisseria*, *H. parainfluenza*, *L. mirabilis*, *R. aeria*, *S*. *australis* and *S. sanguinis* and Increased abundance of *S. parasanguinis* seen in smokers Genera *Aggregatibacter*, *Bergeyella*, *Capnocytophaga*, *Selenomonas*, *Prevotella*, *Porphyromonas*, *Tannerella*, *Parvimonas*, *Filifactor*, *Bacteroidales [G2]* and *Peptostreptococcaceae* was noted in smokers
35	Yadav et al. (31) India, 2023	Saliva	N/A	Alcoholic consumption and vegan diet	N/A	Smokers had higher concentrations of *Streptococcus*, *Prevotella*, *Veillonella* and *Tannerella* and lower concentrations of *Fusobacterium*, *Selenomonas* and *Neisseria* when compared with non-Smokers *Clostridium*, *Filifactor* and *Corynebacterium* were only found in smokers
36	Yu et al. (52) Korea, 2024	Saliva	Overall—20's to 50's	Coffee consumption and Drinking	No difference in alpha diversity between smokers	Abundance of *Oribacterium*, *Atopobium*, and 21 *Megasphaera*, *Eubacterium_nodatum_group*, *Butyrivibrio* were higher in smokers

### 3.3 Differences in the abundance of various taxa between smokers and non-smokers

*Firmicutes* were identified as the most abundant phylum across most studies compared to other types of phyla, including *Proteobacteria*, *Bacteroidetes*, and *Fusobacteria*, which varied in abundance among smokers and non-smokers. Four studies reported *Fusobacteria* being depleted in non-smokers and higher in smokers (18, 22, 27, 49). In contrast, *Fusobacteria*, in particular, was lower in smokers and drinkers and more abundant in the control group (42). Studies conducted using an oral rinse and saliva of cigarette and tobacco smokers were enriched with phylum *Actinobacteria* in current users (25, 28, 46, 49). Similarly, an abundance of *Actinobacteria* in water pipe smokers was reported in another study (36). *Bacteroidetes* dominated smoker and chewer samples in two studies (22, 29) compared to another, which reported a lower relative abundance in cigarette smokers compared to e-cigarette users and controls (23). In terms of genera, most studies reported different types of genera in various types of samples from smokers and healthy controls. *Streptococcus* was relatively reported higher in abundance in smokers in several studies. *Prevotella* and *Veillonella*, mostly independently, were also found as predominant genus in tobacco users (21, 24, 31, 34, 37, 38, 42–44, 49, 51), while another data reported a significant depletion in the saliva and supragingival plaques of smokeless tobacco users (50). *Neisseria* was also observed to be higher among other genera in smokers in two studies conducted in China and Denmark (38, 48). The detailed findings are presented in Table 2.

### 3.4 Differences in metabolic pathways between smokers and non-smokers

Among the 36 studies included, only 9 of them explored the differences in metabolic pathways (15, 19, 26, 27, 30, 37, 39, 40, 49). Wu et al. reported that xenobiotic biodegradation, amino acid metabolism pathways, glycan biosynthesis, and metabolism were enriched in smokers. Further pathways related to aerobic metabolism [tricarboxylic acid (TCA) cycle, oxidative phosphorylation and nitrate reduction] were depleted in current smokers (19, 49). Similarly, Sato et al. also reported significant differences in pathways related to the TCA cycle, glyoxylate cycle, and several compound biosynthesis and degradation between smokers and non-smokers (40). Jia et al. reported that acid production, amino acid-related enzymes and amino sugar, and nucleotide sugar metabolism were all enriched in smokers (37). A recent study on cigarette smokers reported depletion of pathways related to membrane transport and lipid metabolism in smokers as well as xenobiotics biodegradation and enrichment of pathways related to the metabolism of amino acids, nucleotides, vitamins, terpenoids, polyketides, and glycans (26). In the case of smokeless tobacco users, Srivastava et al. reported an increase in amino acid metabolism, xenobiotic biodegradation, and cellular process and signaling (27). Another study also reported an increase in pathways related to amino acid metabolism, synthesis, and degradation (15). Moreover, Sawant et al, observed an increase in pathways related to reductive TCA cycle and pyrimidine biosynthesis in chewing tobacco users (30).

### 3.5 Methodological quality of the studies

The quality of the studies can be found in Supplementary Table S1. Five studies were graded as very good; twenty-six articles were of good quality, whereas the rest of them were of satisfactory quality.

## 4 Discussion

This review aimed to evaluate the available evidence on the impact of the use of tobacco in various forms on healthy humans' oral microbiomes. To our knowledge, this is the first systematic and comprehensive review that summarizes the impact of tobacco use on the oral microbiome. Although there were variations in design, quality of the studies, and characteristics, our results highlight that smoking, regardless of the form, altered the normal equilibrium of the oral microbiome. This evidence is in accordance with previous results obtained analyzing oral microbiomes in culture methods and animal models (53, 54). Despite the limited number of studies, other less-known forms of smoking also seemed to be associated with changes in the oral microbiome.

The current review of data from clinical studies emphasizes that cigarette smoking is found to cause alteration in the oral bacterial profiles. *Streptococcus* was notably a predominant genus in most studies. In healthy populations, streptococci are common members of the subgingival and supragingival habitats and are early commensal invaders of these environments. However, these commensals have been shown to inhibit the proinflammatory response, which is how they predominantly modulate the immune system and aid in biofilm development (55). Notably, the majority of the other bacteria that were significantly increased in smokers were anaerobes, including *Prevotella* and *Veillonella*. This could be related to the deprivation of oral oxygen due to cigarette smoking. Smoking may create a depletion of an oxygen environment in the mouth. It would reflect on the oxygen availability of microbes in the oral cavity, leading to the oral microbial ecology alteration. These were also reported to increase smokers' gut microbiome (47, 56). *Veillonella* and *Actinomyces* were the anaerobic bacteria found to be higher in smokers, and these could promote the development of biofilms in the oral cavity (37). Interestingly, *Actinomyces* have also been enriched in several cancers, including liver, esophagus, colorectal cancer, etc. (57–60). *Actinomyces* has been shown to the production of various immunological and microbial-related genes, such as TLR2, TLR4, and NF-B, which support the growth of colorectal cancer by controlling inflammation by activating the downstream TLR4/NF-B pathway (60). *Actinomyces* also has been shown to modulate the presence of several other gram-negative bacteria (60). It also reduces antitumor immunity by preventing CD8+ T cell invasion in colorectal cancer (60). Furthermore, nitrate in vegetables is often converted to oral nitrate, which has the potential to make the oral cavity more acidic, and anaerobic bacteria, especially *Actinomyces* and *Veillonella*, promote this conversion (61, 62). This acidic environment has been shown to encourage the growth of biofilms and is linked to oral cavity diseases (63). Decreased local oxygen tension and acidic environment are also likely to promote periodontal anaerobes *Fusobacterium*, *Treponema*, and *P. gingivalis*, which are implicated in the development of periodontitis (64).

The oral cavity is often the first contact with smoke and hence may play an essential role in the degradation of toxic compounds. The depletion of several biodegradation pathways in current smokers suggests potential downstream consequences. A key observation in smokers was the enriched degradation of polycyclic aromatic hydrocarbons and other constituents in cigarette smokers (19). Amino acid-related enzymes and amino sugar and nucleotide sugar metabolism were notably abundant in smokers compared to non-smokers (37). Alternatively, these toxic compounds may saturate the enzymes responsible for their degradation, thus killing the bacteria possessing these enzymes (19). The toxic components in cigarette smoke have been shown to alter the oral immune response, and it has been implicated in the pathogenesis of several oral diseases, including periodontitis and oral cancer (8, 64).

Oral epithelial cells actively participate in oral immune response by expressing specific receptors, including toll-like receptors (TLRs). TLRs are receptors in immune response expressed by cell surfaces and internal vesicles and their stimulation lead to activation multiple intracellular signaling cascades (65) One of the main downstream signaling cascades is the NF-KB, a critical transcription factor that encourages the expression of chemokines, cytokines, and co-stimulatory and adhesion molecules (66). Cigarette smoke has been shown to increase the expression of and alter the functional activation of these receptors, including TLR-2, TLR-4, and others (67, 68). Interestingly, the taxa reported to be enriched in smokers including *Fusobacteria*, *Veillonella*, *Prevotella*, and *Actinomyces*, as well as other microorganisms, also bind to TLR-2 and TLR-4 using their peptidoglycan and lipopolysaccharide cell walls, and these TLR-2 or TLR-4 mediated signaling leads to up-regulation of several proinflammatory pathways (69–74). TLRs and their signaling machinery have been subsequently implicated in a wide range of human diseases, including several cancers, especially oral cancers (75–77).

Tobacco components have also been shown to increase the virulence of specific periodontal pathogens, particularly for *P. gingivalis*, which has multiple virulence factors (64, 78, 79). Oxidative stress-related proteins in *P. gingivalis* are up-regulated in the presence of nicotine and other products, which helps in adaptability and survival ability in a low-oxygen environment and biofilms (78, 80). *P. gingivalis* biofilms have reduced proinflammatory properties, which can help enhance sustainability (80, 81). However, it was interesting to note that the upregulation of *P. gingivalis* was reported by two published studies only. *P. gingivalis* is also known to facilitate many microbial colonizers, including *S. oralis*, *Streptococcus gordonii*, *Actinomyces viscosus*, *Fusobacterium* spp *& Prevotella intermedia* (79, 82–84), which has been reported to be upregulated by multitude of studies included in the review.

Interestingly, one of the studies reported that the overall oral microbiome composition of former smokers did not differ in comparison to never smokers; this indicates that changes in the oral microbiome influenced by smoking are permanent (19). Such findings are encouraging and can lay the foundation for microbiome-targeted approaches for smoking cessation and disease prevention.

In our review, we noticed that only very few studies have explored the impact of use of shisha or waterpipe on the oral microbiome. It is now known that waterpipe smoke constitutes many of the same toxicants and is associated with the risk of disease (36). Relative to water pipe smoking, out of the four studies included, *Streptococcus sanguinis* was found to be higher in smokers (35). Overall, phyla *Firmicutes* was the most abundant phylum in those combined with other forms of tobacco smoking such as medwakh and cigarettes (35, 36). Few of these bacterial species are known to be a common cause of human respiratory diseases and infections, notably where tobacco consumption is a significant risk factor (85, 86). It is pretty unclear as to what specific bacteria taxa are associated with water pipes due to the scarcity of resources available; however, this could be mainly influenced by the habits of the subjects and other exposures as well.

Smokeless tobacco can also impact oral microbiota, increasing the risk for oral disease pathologies. Due to the nicotine concentration in smokeless tobacco, the growth of *S.mutans* places the user at an increased risk for dental caries (87). Hung et al. suggested that these tobacco products can increase caries development by fostering *S.mutans* formation on tooth surfaces (88). Further, streptococci species are known to produce acetaldehyde. Acetaldehyde, a carcinogenic compound, production has been proposed as a mechanism by which bacteria can contribute to oral carcinogenesis (34). This is supported by abundant levels of *Streptococcus* genera that indicated alterations in smokeless tobacco users compared to controls (15, 27). Furthermore, *Fusobacteria* abundant in smokeless tobacco users is an opportunistic pathogen and has been known to be capable of growth in acidic conditions (15). *Fusobacteria* has reportedly been noted in human colorectal carcinoma, suggesting it may have originated from the oral cavity. They promote tumor development by inducing inflammation and the immune response of the host to produce inflammatory factors (89). In addition, these species have reportedly been found to be abundant in head and neck cancer samples (90).

This review noted that sample collection sites in the oral cavity subsequently differed within the studies. This site variation could produce significant bias as the sites may vary in microbial composition. For instance, salivary samples may reflect the bacteria shed from the total oral cavity, whereas tissue sampling would be a deeper representation of the microbiome concerning the host (91). Hence, it wouldn't be rational to assume the impacts of smoking caused by components of tobacco smoke are similar across all microenvironments (44). Further studies are recommended to elucidate the different ecology of these environments, as interpreting the data of a mixture of sample types may obscure meaningful associations and patterns.

The current review highlights that the studies reported until now relied on genetic characterization of the microbiome using 16S sequencing methodology without adequate examination of this functionality. Only three studies employed shotgun sequencing (28, 33, 44). Given that shotgun metagenomic sequencing provides better strain level resolution and functional insights, the field should focus more on this sophisticated methodology, in combination with metabolomics and metaproteomic, in decoding host-microbiome interactions. Microbiome architecture can be highly varied among humans, with inter-individual variation presenting a substantial challenge, necessitating the development of sophisticated machine learning processes that predict the impact of microbiome and metabolites on physiological and pathological situations. Despite these constraints, understanding the ubiquitous activities of microbially regulated metabolites can open up a new avenue for enhancing oral health. One of the potential clinical implication of deciphering host-microbial interactions would be management strategies for tobacco-related illnesses, including smoking cessation strategies by altering the microbiota with probiotics, prebiotics, and other related methods. There is currently insufficient data despite the possibility that several preventive and therapeutic applications might be effective in theory. These are primarily related to the possibility of eubiosis being restored upon smoking cessation. As a matter of fact, we have uncovered a dearth of research on this aspect considering the abundance of studies on tobacco use and oral microbiota and needs to be explored further.

One of the limitations of the current review is the heterogeneity in the methods and the outcome reporting in the included studies, which hindered comparability and quantitative analysis. However, this is a common limitation reported by most of the reviews on microbiome, because of the inherent heterogeneity in the methodology. Further, we have included articles published only in the English language.

## 5 Conclusion

In this review, it is majorly observed that smoking and smokeless tobacco influence the oral microbial community composition, and there is a definitive shift in the abundance of oral taxa favoring an anaerobic environment, thus promoting a proinflammatory milieu. It is suggested that smoking may perturb the balance of the oral microbiome by affecting the relationships between bacteria and altering their metabolic pathways. However, smokeless and smoking tobacco are a mixture of multiple toxicants, and their direct impact on the oral microbiome is yet unclear. The effect of tobacco on microbial metabolism needs to be elucidated and is critical to our understanding of the etiology of oral and systemic diseases, as oral microbial dysbiosis are associated with several systemic conditions.

## Data Availability

The original contributions presented in the study are included in the article/Supplementary Material, further inquiries can be directed to the corresponding author.
