# Supplementary material for: Effect of different forms of tobacco on the oral microbiome in healthy adults: a systematic review
Source: Front Oral Health. 2024 Feb 20;5:1310334. doi: 10.3389/froh.2024.1310334 (PMC10912582; doi:10.3389/froh.2024.1310334)
Supplement: Supplementary file 1 [file Table4.docx]

**Supplementary Table 4: Newcastle Ottawa Scale for quality of studies**

| No | Author, country, year | Selection | | | | Comparability | Outcome | | Overall |
| --- | --- | --- | --- | --- | --- | --- | --- | --- | --- |
|  |  | Representativeness of the sample | Samples size | Non respondents | Ascertainment of the exposure | Confounding factors are controlled | Assessment of outcome | Statistics |  |
| 1 | Thomas et al, Brazil, 2014 | * | * |  | * | * | * | * | 5 (Satisfactory) |
| 2 | Mason et al, USA, 2015 | * | * | * | * |  | ** | * | 7 (good) |
| 3 | Wu et al, USA, 2016 | * | * | * | * |  | ** | * | 7 (good) |
| 4 | Hernandez et al, USA, 2017 | * | * |  | * | * | * | * | 5 (Satisfactory) |
| 5 | Yu et al, USA, 2017 | * | - | * | * | ** | ** | * | 8 (good) |
| 6 | Rodríguez- Rabassa et al, USA, 2018 | * | * |  | * | * | * | * | 5 (Satisfactory) |
| 7 | Stewart et al, USA, 2018 | * |  | * | * |  | * | * | 5 (Satisfactory) |
| 8 | Vallès et al, UAE, 2018 | * | * | * | ** |  | * | * | 7 (good) |
| 9 | Beghini et al, USA, 2019 | * | * | * | * | * | * | * | 7 (good) |
| 10 | Lin et al, USA, 2019 | * | * | * | * | * | * | * | 7 (good) |
| 11 | Yang et al, USA, 2019 | * | * | * | * | * | * | * | 7 (good) |
| 12 | Al Bataineh et al, UAE, 2020 | * | * | * | ** |  | ** | * | 8 (good) |
| 13 | Al- Zyoud et al, Jordan, 2020 | * | * |  | * |  | ** | * | 6 (satisfactory) |
| 14 | Halboub et al, UAE, 2020 | * | * | * | * | * | * | * | 7 (good) |
| 15 | Sato et al, Japan, 2020 | * | * | * | * | ** | ** | * | 9 (very good) |
| 16 | Wirth et al, Hungary, 2020 | * | * | * | ** | ** | ** | * | 10 (very good) |
| 17 | Bašić et al, Croatia, 2021 | * | * | * | * | * | * | * | 7 (good) |
| 18 | Al Kawas et al, UAE, 2021 | * | * |  | ** |  | ** | * | 7 (good) |
| 19 | Jia et al, China, 2021 | * | * | * | * |  | ** | * | 7 (good) |
| 20 | Li et al, China, 2021 | * | * | * | * | * | * | * | 7 (good) |
| 21 | Srivastava et al, India, 2021 | * | * | * | * | * | ** | * | 8 (good) |
| 22 | Wu et al, Iran, 2021 |  |  |  |  |  |  |  |  |
| 23 | Al-Marzooq et al, UAE, 2022 | * | * |  | ** |  | ** | * | 7 (good) |
| 24 | Gopinath et al, India, 2022 | * | * | * | ** |  | ** | * | 8 (good) |
| 25 | Pfeiffer et al, Germany, 2022 | * | * | * | * | * | * | * | 7 (good) |
| 26 | Poulsen et al, 2022, Denmark | * | * | * | * | * | * | * | 7 (good) |
| 27 | Sharma, 2022, India | * | * | * | * | * | * | * | 7 (good) |
| 28 | Suzuki et al, Japan, 2022 |  |  |  |  |  |  |  |  |
| 29 | Antonello et al, Italy, 2023 | * | * | * | * | ** | ** | * | 9 (very good) |
| 30 | Bahuguna et al, India, 2023 | * | * |  | ** |  | ** | * | 7 (good) |
| 31 | Huang et al, China, 2023 | * | * |  | ** |  | ** | * | 7 (good) |
| 32 | Sami, Sudan, 2023 | * | * |  | ** |  | ** | * | 7 (good) |
| 33 | Sawant et al, India, 2023 | * | * |  | ** |  | ** | * | 7 (good) |
| 34 | Galvin et al, Ireland, 2023 | * | * | * | * | ** | ** | * | 9 (very good) |
| 35 | Yadav et al, India, 2023 | * | * |  | ** |  | ** | * | 7 (good) |
| 36 | Yu et al, Korea, 2024 | * | * | * | * | ** | ** | * | 9 (very good) |
