# Supplementary material for: Effect of different forms of tobacco on the oral microbiome in healthy adults: a systematic review
Source: Front Oral Health. 2024 Feb 20;5:1310334. doi: 10.3389/froh.2024.1310334 (PMC10912582; doi:10.3389/froh.2024.1310334)
Supplement: Supplementary file 2 [file Datasheet1.docx]

| **Search No.** | **Searches** | **Hits** |
| --- | --- | --- |
| 1 | smok*.ti,ab. | 534116 |
| 2 | tobacco*.ti,ab. | 183009 |
| 3 | cigarette*.ti,ab. | 129551 |
| 4 | shisha*.ti,ab. | 543 |
| 5 | hookah*.ti,ab. | 1476 |
| 6 | e-cig*.ti,ab. | 11049 |
| 7 | (vape* or vaping*).ti,ab. | 5277 |
| 8 | water$pipe*.ti,ab. | 1444 |
| 9 | microbiome*.ti,ab. | 65798 |
| 10 | microbial*.ti,ab. | 352380 |
| 11 | microbiota*.ti,ab. | 110428 |
| 12 | bacteria*.ti,ab. | 1254878 |
| 13 | microflora*.ti,ab. | 28705 |
| 14 | 16s rRNA gene*.ti,ab. | 65307 |
| 15 | metagenome*.ti,ab. | 9619 |
| 16 | (mouth* or oral*).ti,ab. | 1319055 |
| 17 | 1 or 2 or 3 or 4 or 5 or 6 or 7 or 8 | 644099 |
| 18 | 9 or 10 or 11 or 12 or 13 or 14 or 15 | 1561774 |
| **19** | **16 and 17 and 18** | **1323** |

Table S1: Search strategy in MEDLINE (Ovid)

Table S2: Search strategy in CINAHL Database

| **Search No.** | **Searches** | **Hits** |
| --- | --- | --- |
| 1 | TI smok* OR AB smok* | 110373 |
| 2 | TI tobacco* OR AB tobacco* | 36181 |
| 3 | TI cigarette* OR AB cigarette* | 28059 |
| 4 | TI shisha* OR AB shisha* | 135 |
| 5 | TI hookah* OR AB hookah* | 714 |
| 6 | TI e-cig* OR AB e-cig* | 4984 |
| 7 | TI vape* OR AB vape* | 754 |
| 8 | TI vaping* OR AB vaping* | 1917 |
| 9 | TI water*pipe* OR AB water*pipe* | 557 |
| 10 | TI microbiome* OR AB microbiome* | 7621 |
| 11 | TI microbial* OR AB microbial* | 13008 |
| 12 | TI microbiota* OR AB microbiota* | 11970 |
| 13 | TI bacteria* OR AB bacteria* | 55007 |
| 14 | TI microflora* OR AB microflora* | 1233 |
| 15 | TI 16s rRNA gene* OR AB 16s rRNA gene* | 1475 |
| 16 | TI metagenome* OR AB metagenome* | 168 |
| 17 | TI mouth* OR AB mouth* | 18856 |
| 18 | TI oral* OR AB oral* | 169481 |
| 19 | S1 OR S2 OR S3 OR S4 OR S5 OR S6 OR S7 OR S8 OR S9 | 130308 |
| 20 | S10 OR S11 OR S12 OR S13 S14 OR S15 OR S16 | 73632 |
| 21 | S17 OR S18 | 182906 |
| **22** | **S19 AND S20 AND S21** | **228** |

Table S3: Search strategy in Web of Science

| **Search No.** | **Searches** | **Hits** |
| --- | --- | --- |
| 1 | TI=smok* OR AB=smok* | 359126 |
| 2 | TI=tobacco* OR AB=tobacco* | 135197 |
| 3 | TI=cigarette* OR AB=cigarette* | 84704 |
| 4 | TI=shisha* OR AB=shisha* | 595 |
| 5 | TI=hookah* OR AB=hookah* | 1190 |
| 6 | TI=e-cig* OR AB=e-cig* | 8936 |
| 7 | TI=vape* OR AB=vape* | 1764 |
| 8 | TI=vaping* OR AB=vaping* | 3622 |
| 9 | TI=water*pipe* OR AB=water$pipe* | 1127 |
| 10 | TI=microbiome* OR AB=microbiome* | 63089 |
| 11 | TI=microbial* OR AB=microbial* | 381512 |
| 12 | TI=microbiota* OR AB=microbiota* | 100249 |
| 13 | TI=bacteria* OR AB=bacteria* | 1019065 |
| 14 | TI=microflora* OR AB=microflora* | 22526 |
| 15 | TI=16s rRNA gene* OR AB=16s rRNA gene* | 68249 |
| 16 | TI=metagenome* OR AB=metagenome* | 8580 |
| 17 | TI=mouth* OR AB=mouth* | 113417 |
| 18 | TI=oral* OR AB=oral* | 772073 |
| 19 | #1 OR #2 OR #3 OR #4 OR #5 OR #6 OR #7 OR #8 OR #9 | 452624 |
| 20 | #10 OR #11 OR #12 OR #13 OR #14 OR #15 OR #16 | 1352106 |
| 21 | #17 OR #18 | 867550 |
| **22** | **#19 AND #20 AND #21** | **884** |
